# Comparative growth kinetics and drug susceptibility of *Mycobacterium tuberculosis* lineages prevalent in Ethiopia: implications for tuberculosis treatment and management

**DOI:** 10.3389/fmicb.2024.1512580

**Published:** 2025-02-04

**Authors:** Tesfaye Gebreyohannis Hailemariam, Melaku Tilahun, Abay Atnafu, Tesfaye Gelanew, Tewodros Tariku Gebresilase, Mekdes Alemu Tola, Abaysew Ayele, Shewki Moga Siraj, Workineh Shibeshi, Kidist Bobosha, Liya Wassie, Yonas Hirutu, Ephrem Engidawork

**Affiliations:** ^1^Department of Pharmacology and Clinical Pharmacy, School of Pharmacy, College of Health Sciences, Addis Ababa University, Addis Ababa, Ethiopia; ^2^Armauer Hansen Research Institute (AHRI), Addis Ababa, Ethiopia; ^3^Ethiopian Public Health Institute, Addis Ababa, Ethiopia

**Keywords:** *Mycobacterium tuberculosis*, sub-lineages, growth kinetics, minimum inhibitory concentrations, optimizing TB treatment

## Abstract

Tuberculosis (TB) remains a global health challenge, with treatment outcomes influenced by the genetic diversity of *Mycobacterium tuberculosis* (*Mtb*) strains. This study examines the growth kinetics and drug susceptibility of *Mtb* strains from different lineages in Ethiopia to understand their impact on disease management. *Mtb* strains, including sub-lineages 4.1.2.1, 4.2.2.2, 4.6.3, lineages 3 and 7, and the reference strain H37Rv (ATCC 27294), were cultured in liquid 7H9 Middlebrook broth. Growth began on day 6 post-inoculation. Sub-lineage 4.1.2.1 showed rapid exponential growth by day 9, reaching the stationary phase by day 15. Sub-lineage 4.1.2.1 followed by sub-lineage 4.2.2.2 had the highest maximum growth concentration (*C*_max_), indicating enhanced growth efficiency and adaptive traits that may increase their pathogenicity or resistance to host defenses or anti-TB drugs. To support this observation, the minimum inhibitory concentrations (MIC) for first-line anti-TB drugs were assessed for all the studied *Mtb* strains using the microdilution broth method. While all strains were susceptible, MIC values varied. Sub-lineages 4.1.2.1 and 4.2.2.2 had MIC values matching WHO’s critical concentrations (except for rifampicin). Lineage 3 showed increased sensitivity to rifampicin, isoniazid, and streptomycin, requiring only half the standard concentration. Lineage 7 also exhibited higher sensitivity to rifampicin and streptomycin. These findings highlight the importance of considering lineage-specific differences in *Mtb* strains for optimizing treatment regimens and improving TB control strategies, particularly in regions with diverse *Mtb* populations like Ethiopia.

## Introduction

Tuberculosis (TB), caused by *Mycobacterium tuberculosis* (*Mtb*), continues to be a critical global health issue, claiming the lives of over 1.6 million people and further causing infections in about 10.6 million individuals in 2021 (Bagcchi, 2023). If left untreated, up to 70% of those afflicted with the disease will not survive (Tiemersma et al., 2011). The vast majority of these cases occur in developing regions, underscoring the impact of poverty, subpar living conditions, and inadequate healthcare (Bai and Ameyaw, 2024).

Mycobacteria in the *Mycobacterium tuberculosis* complex (MTBC) share a strong genetic similarity (99.9%) and identical 16S rRNA sequences but differ in host preferences, disease potential, treatment outcomes, drug resistance, BCG vaccine effectiveness, transmission ease, and epidemiological outbreaks. Insights into these differences are vital for developing new vaccines and managing drug-resistant *Mtb*, ultimately improving treatment strategies and healthcare (Chae and Shin, 2018; Brosch et al., 2002).

The MTBC consists of nine lineages (1 to 9) that have adapted to human hosts (Hailu et al., 2023). These lineages (L) are divided into two clades. The first, “modern” clade, which is adapted to humans and characterized by the TbD1 deletion, forms a monophyletic group (a single phylogenetic lineage) and consists of L-2 (East-Asian), L-3 (East-African Indian), and L-4 (Euro-American) (Moopanar and Mvubu, 2020; Coscolla and Gagneux, 2014). These lineages have undergone more recent diversification than other MTBC strains. Lineages 2 and 4 are not only more globally widespread but also exhibit greater virulence compared to other lineages with more restricted geographical distribution (Coscolla and Gagneux, 2014). The second, ancient clade (are paraphyletic), which retains the TbD1 region, includes lineages adapted to both humans and animals: L-1 (Indo-Oceanic), L-5, L-6 (*M. africanum* West Africa 1 and 2), L-7 (Ethiopia), L-8 (Rwanda) and L-9 (Related to East African lineages) (Moopanar and Mvubu, 2020; Coscolla and Gagneux, 2014; Ngabonziza et al., 2020; Coscolla et al., 2021). Lineages 1, L-2, and L-3 lack RD239, RD105, and RD750 sequences, respectively, while lineage 4 has a deletion in the pks15/1 gene. Lineages 5 and L-6 lack the RD9 region, with lineage 6 also missing RD7, RD8, and RD10. Additionally, lineages 5 and 6 have unique markers, RD711 and RD702 (Moopanar and Mvubu, 2020).

Africa is the only continent where all MTBC lineages are present, with Ethiopia being a major hub for most lineages (Ngabonziza et al., 2020). In a study of 4,371 clinical isolates over 20 years, 99.5% were *Mtb* and 0.5% were *M. bovis*. The proportions of lineages were L4 (62.3%), L3 (21.7%), L1 (7.9%), and L7 (3.4%). The most common sub-lineages were L4.2.2.2 (ETH/SIT149) (14.4%), L4.10/SIT53 (9.7%), L3. ETH1/SIT25 (7.2%), and L4.6/SIT37 (5.5%) (Mekonnen et al., 2019). Lineage 4, especially sublineage 4.2.2.2, is spreading in Ethiopia and is linked to more widespread and multidrug-resistant (MDR) TB infections (Mekonnen et al., 2023), suggesting unique virulence traits.

The outcome of tuberculosis infection and disease varies significantly and has been largely attributed to factors such as host characteristics, MTBC genomic diversity, and environmental influences. Different MTBC strains exhibit variations in virulence and immunogenicity, and there is growing evidence suggesting that strain diversity may play a role in human TB. However, the extent to which MTBC genomic diversity impacts the progression of disease in clinical settings requires further research (Coscolla and Gagneux, 2010).

Generating data on the minimum inhibitory concentration (MIC) and growth kinetics of the different *Mtb* strains contribute to improving TB treatment strategies and enhancing the dynamics of healthcare system. This study attempted to optimize growth kinetics for most prevalent MTBC lineages circulating in Ethiopia, by measuring colony-forming units (CFU), turbidity, and optical density at 600 nm (OD_600_). The data provide valuable insights into growth rates and minimum inhibitory concentrations (MIC), thereby generating evidence for potential personalized TB treatment options.

## Materials and methods

### Study setting

This was an experimental study, where stored *Mtb* isolates, collected from active pulmonary TB patients as part of the TBGEN study, were used. Five clinically identified *Mtb* strains, with known genetic characteristics (using whole-genome sequencing, and phenotypic traits, such as drug resistance profiles) along with a reference *Mtb* strain, H37Rv (ATCC 27294), were selected from the Armauer Hansen Research Institute (AHRI) TB laboratory. The samples were selected based on high genome sequence quality, representing various *Mtb* lineages and sub-lineages circulating in Ethiopia.

### Whole genome bioinformatic analyses

Raw read quality was assessed with FASTQC (v0.12.1) (Yang et al., 2013). Trimmomatic (v0.39) was used to remove low-quality reads, short fragments, and adapter sequences (Daniyarov et al., 2020), the cleaned reads were then aligned to the H37Rv reference genome (NC 000962.3) using BWA (v0.7.18) (Li, 2013). Single nucleotide polymorphisms (SNPs) were identified using the bcftools (v1.8) (Phelan et al., 2019). TBProfiler (v6.2.0) was used to predict drug resistance profiles and lineages (Verboven et al., 2022). SNP-based phylogenetic trees were constructed using IQ-TREE (v2.0.3) with a maximum likelihood model and 1,000 bootstrap replicates, and subsequently annotated and visualized with interactive Tree of Life (iTOL) (v6.9) (Yu, 2020).

### *Mycobacterium tuberculosis* growth kinetics

To ensure all strains started at a similar growth phase, all *Mtb* clinical and H37Rv (ATCC 27294) strains standardized concentration of 100 μL (OD_600_ of 0.2) and each having two replicates, were cultured on 10 mL Middlebrook 7H9 broth (Difco, Detroit, MI, United States) supplemented with 10% OADC (oleic acid, albumin, dextrose, and catalase; Becton Dickinson and Company, Sparks, MD, United States), 0.2% glycerol, and 0.05% Tween 80. The inoculated cultures were incubated in an incubator (Panasonic, MCO-801C-PE) at 37°C for 21 days. Within the first 24 h after inoculation, the culture was visually examined to detect any signs of contamination (Sarkar et al., 2012).

To measure colony-forming unit concentration (CFU/mL), a 100 μL bacterial suspension (from stock vials with an OD_600_ of 0.2) was used to prepare serial dilutions, which were then inoculated in duplicate onto Middlebrook 7H11 agar plates (Difco, Detroit, MI, United States) containing 0.5% glycerol and 10% OADC growth enrichment (Becton Dickinson, Cockysville, MD, United States). During the 21-day incubation, turbidity, CFU/mL, and OD_600_ measurements were regularly taken every 3 days (Sarkar et al., 2012). The CFU/mL results were systematically correlated with McFarland standard data and OD_600_ readings. The optical density at 600 nm (OD_600_) of the bacterial suspensions was measured using a BioSpectrophotometer (Eppendorf, AG, 22331), while the turbidity of the suspensions was evaluated through nephelometry (DEN-1). The *Mtb* colonies were carefully counted and adjusted according to the dilution factor.

### *Mycobacterium tuberculosis* minimum inhibitory concentration

The MICs were determined as the lowest drug concentration after twofold serially diluted concentration of the drugs that inhibits growth of more than 99.0% of a bacterial proportion of the tested *Mtb* strains, on suitable broth method, within 14 to 21 days of incubation at 37°C (Schönfeld et al., 2012). MIC of the first-line anti-tuberculosis (TB) drugs isoniazid (INH), rifampicin (RIF), ethambutol (EMB), and streptomycin (STR) was determined using the microdilution broth method (Schön et al., 2021). *Mtb* clinical isolates were initially cultured and adjusted to a turbidity matching the 1.0 McFarland (McF) standard, which is approximately 1.97 × 10^6^ CFU/mL. This was done by suspending bacterial colonies in sterile saline and visually comparing the turbidity to the McFarland standard. The bacterial suspension was then diluted in Middlebrook 7H9 broth to achieve the final inoculum density required for the assay (Peñuelas-Urquides et al., 2013).

Serial dilutions of each drug were prepared in sterile 96-well microtiter plates, starting from concentration ranges based on critical thresholds: 0.1 μg/mL for INH, 1 μg/mL for RIF, 5 μg/mL for EMB, and 2 μg/mL for STR. MIC determination assays were carried out in triplicate. Each well was inoculated with the 10 μL bacterial suspension, and the plates were incubated at 37°C for 7 to 14 days, depending on the strain’s growth rate (Santos et al., 2020). A growth control (without drug) and a sterile control (without inoculum) were included in each plate. The GCs consist of 1:100 dilution of the 10^−2^ inoculum of 1 McF (i.e., 1% of the inoculum present in antibiotic contain wells; GC 1%), and the same inoculum (10^−2^) suspension of 1 McF (i.e., 100% of the inoculum present in antibiotic containing wells; GC100%) (Wikler, 2006).

The MIC was defined as the lowest drug concentration that inhibited visible bacterial growth, indicated by the absence of turbidity in the well. Results were interpreted based on the susceptibility breakpoints established by the World Health Organization’s technical guidelines for microdilution broth method.

### Data analysis

Data were analyzed using GraphPad Prism version 10.00 for Windows, GraphPad Software, www.graphpad.com. Two-way ANOVA was performed to evaluate the growth curves and growth rates of *Mtb* strains and correlation between turbidity, CFU/mL, and OD_600_, followed by Tukey’s multiple comparisons test to determine the differences in growth rates between each strain. An adjusted *p*-value threshold of <0.05 was set for significance.

### Ethical clearance

This study received approval from the Institutional Review Board (IRB) of the College of Health Sciences, Addis Ababa University (Approval No. 072/21/Sop). This study was also approved by the AHRI institutional ethics review committee (P031/18).

## Results

### *Mycobacterium tuberculosis* strains

**Figure 1:**
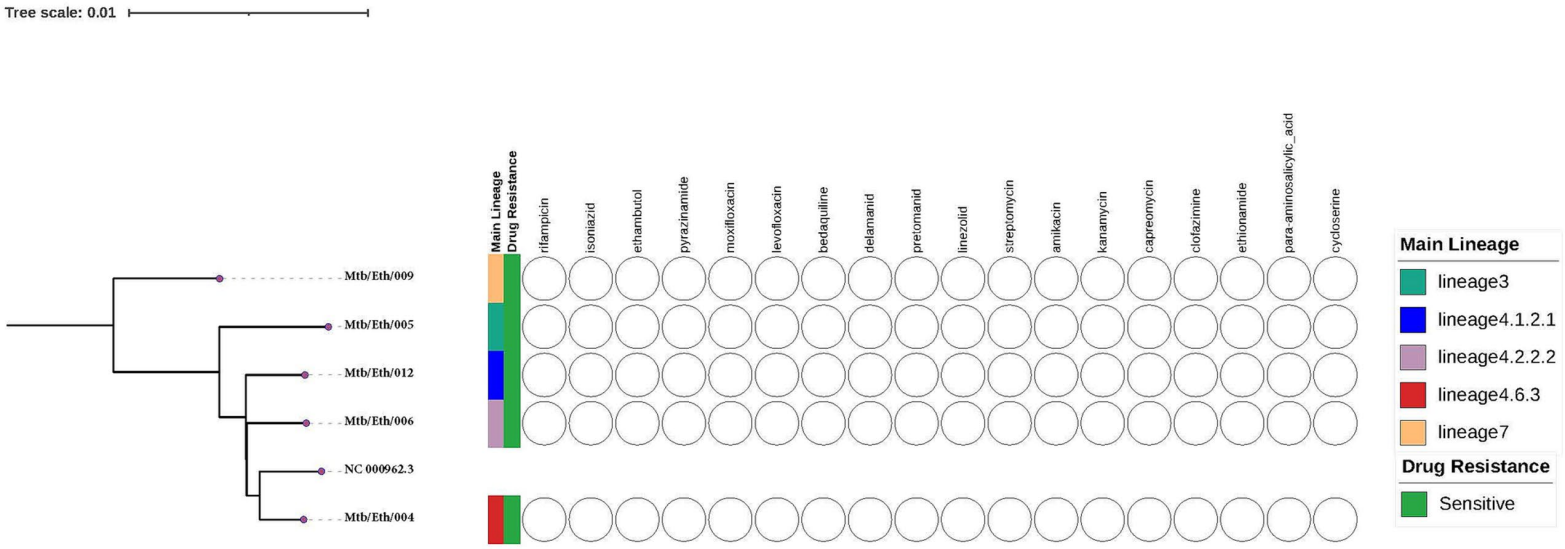
The whole-genome phylogeny and phenotypic drug sensitivity/resistance profiles of the *Mycobacterium tuberculosis* isolates in this study were assessed against standard first and second line anti-TB drugs. The phylogenetic tree was constructed using the H37Rv reference genome (NC 000962.3) obtained from NCBI.

Clinical isolates of *Mtb* used in this study were obtained from a previously characterized TB patients from the TBGEN study. A total of five *Mtb* clinical isolates, with one lineage 3, one lineage 7 and three lineage 4 isolates (sub-lineages 4.2.2.2, 4.6.3, and 4.1.2.1), and one control, H37Rv (ATCC 27294) strain were used. All samples were confirmed to be both genetically and phenotypically sensitive to all first line anti-TB drugs. Additionally, all strains were genetically susceptible to second-line antituberculosis drugs (Figure 1).

### Correlation between *Mtb* concentration measurement methods

**Figure 2:**
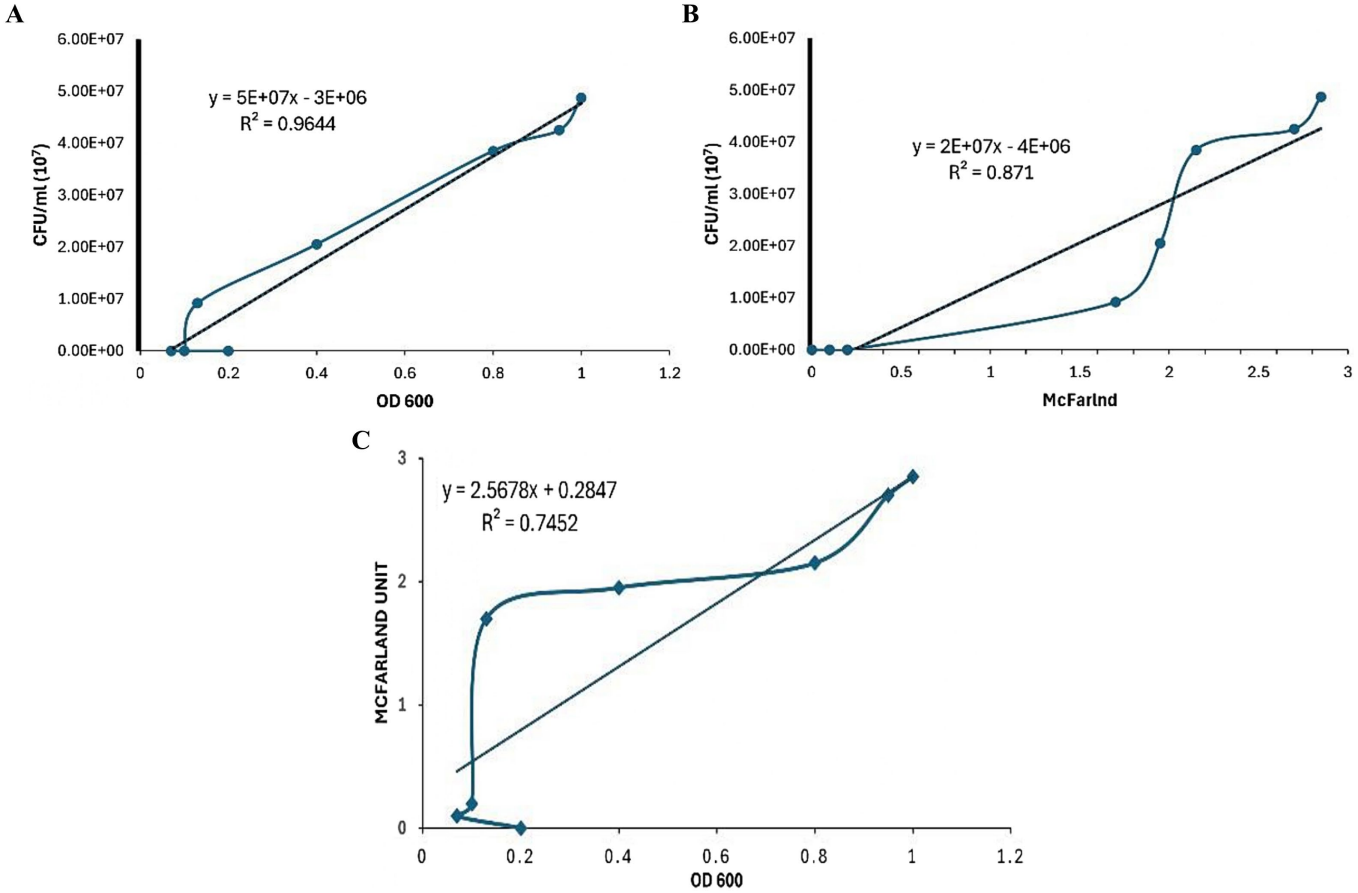
Comparison of the correlation between various *Mycobacterium tuberculosis* concentration methods. **(A)** Correlation between colony-forming units (CFU) per mL and optical density at 600 nm (OD_600_). **(B)** Correlation between colony-forming units (CFU) per mL and McFarland standard. **(C)** Correlation between McFarland standard and optical density at 600 nm (OD_600_).

A strong correlation was observed between CFU/mL and OD_600_ measurements for all clinical isolates and H37Rv *Mtb* samples, with an *R*^2^ value of 0.9644 (Figure 2A). The correlation between CFU/mL and the McFarland standard, and OD_600_ and the McFarland standard were also strong across all samples (*R*^2^ = 0.871 and 0.7452, respectively) (Figures 2B,C).

### *Mtb* growth kinetics

**Figure 3:**
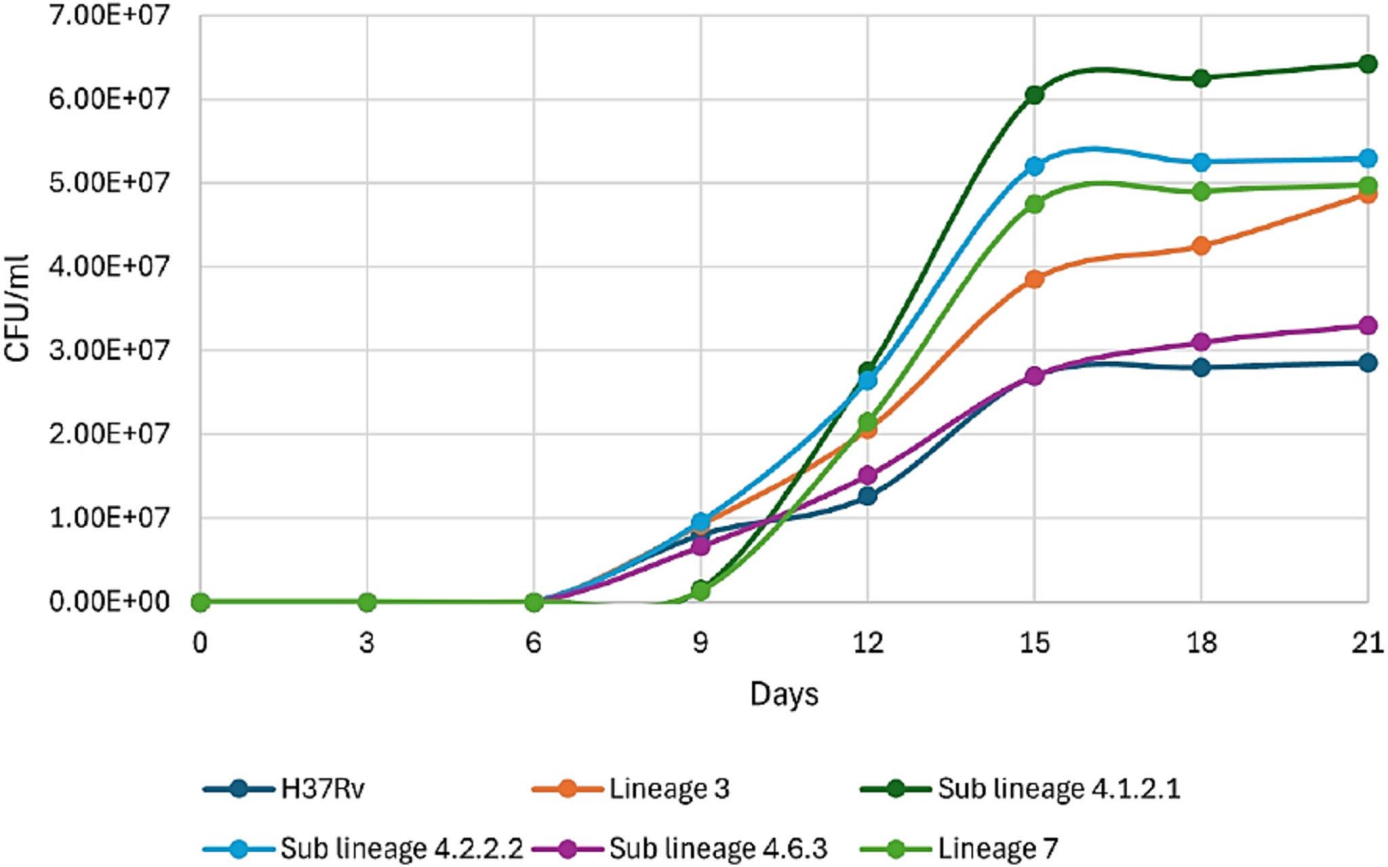
Growth kinetics of different clinical strains of *Mycobacterium tuberculosis* prevalent in Ethiopia, along with H37Rv (ATCC 27294).

*Mycobacterium tuberculosis* strains from various *Mtb* lineages were cultured in liquid 7H9 Middlebrook broth to assess growth rates across lineages prevalent in Ethiopia. All *Mtb* strains showed growth from 6 day onwards (Figure 3). Sub-lineage 4.1.2.1 and lineage 7 began growing on the 9th day, sub-lineage 4.1.2.1 displaying rapid kinetics (exponential phase) over the following 6 days (Figure 3). All *Mtb* strains started to subside their growth and entered the stationary phase since day 15 (Figure 3). A comparison of C_max_ (the maximum point on the growth curve) among different sub-lineages indicate that sub-lineage 4.1.2. 1exhibited a higher *C*_max_, or greater bacterial concentration, than sub-lineage 4.2.2.2, lineage 7, lineage 3, sub-lineage 4.6.3, and H37Rv.

### Multiple comparison on growth rate difference

Sub-lineage 4.2.2.2 exhibited a significant difference in growth rate or CFU compared to all other study strains from day 9 or day 12 until the end of the study (Table 1). Similarly, sub-lineage 4.1.2.1 showed a significant difference in growth rate or CFU compared to H37Rv, sub-lineage 4.2.2.2, and sub-lineage 4.6.3 starting from day 9 onward (Table 2). Other sub-lineages demonstrated inconsistent but significant differences in growth rate or CFU when compared to each other on various study days. No significant differences in growth rate or CFU was observed between H37Rv and sub-lineage 4.6.3 throughout the study period (Table 1).

**Table 1:** Minimum inhibitory concentration for the first-line anti-TB drugs across all *Mycobacterium tuberculosis* strains, along with critical concentration for the microdilution broth method.

First line ant-TB drugs	Minimum inhibitory concentration (μg/mL)						
	Sub-lineage 4.1.2.1	Sub-lineage 4.2.2.2	Sub-lineage 4.6.3	Lineage 3	Lineage 7	H37Rv	Critical concentration
INH	0.1	0.1	0.1	0.05	0.1	0.1	0.1
RIF	0.5	0.5	0.5	0.25	0.25	0.5	1
EMB	4	4	4	4	4	4	5
STM	2	2	1	1	1	1	2

**Table 2:** Tukey’s multiple comparisons test for different *Mycobacterium tuberculosis* sub-lineages growth, as measured by CFU, over study period.

Tukey’s multiple comparisons test	Adjusted *p*-values					
	Day 6	Day 9	Day 12	Day 15	Day 18	Day 21
H37rv vs. sub-lineage 4.1.2.1	ns	ns	0.0001	0.0001	0.0001	0.0001
H37rv vs. sub-lineage 4.2.2.2	ns	0.0105	0.0001	0.0001	0.0001	0.0001
H37rv vs. sub-lineage 4.6.3	ns	ns	ns	ns	ns	ns
H37rv vs. lineage 3	ns	ns	0.0009	0.0001	0.0001	0.0001
H37rv vs. lineage 7	ns	0.0075	0.0002	0.0001	0.0001	0.0001
Sub-lineage 4.1.2.1 vs. sub-lineage 4.2.2.2	ns	0.0007	ns	0.0004	0.0001	0.0001
Sub-lineage 4.1.2.1 vs. sub-lineage 4.6.3	ns	ns	0.0001	0.0001	0.0001	0.0001
Sub-lineage 4.1.2.1vs. lineage 3	ns	ns	ns	0.0001	0.0001	ns
Sub-lineage 4.1.2.1 vs. lineage 7	ns	0.0005	ns	ns	ns	ns
Sub-lineage 4.2.2.2 vs. sub-lineage 4.6.3	ns	ns	0.0001	0.0001	0.0001	0.0001
Sub-lineage 4.2.2.2 vs. lineage 3	ns	0.0015	0.005	0.0001	0.0001	0.0001
Sub-lineage 4.2.2.2 vs. lineage 7	ns	ns	0.0219	0.0001	0.0001	0.0001
Sub-lineage 4.6.3 vs. lineage 3	ns	ns	0.0477	0.0001	0.0001	0.0001
Sub-lineage 4.6.3 vs. lineage 7	ns	ns	0.012	0.0001	0.0001	0.0001
Lineage 3 vs. lineage 7	ns	0.001	ns	0.0001	0.0103	ns

ns, not significant difference of bacterial concentration.

### Minimum inhibitory concentration

The study measured the MIC for first-line anti-TB drugs across all strains using microdilution broth method. Although all strains were susceptible to these drugs, MIC values varied between strains (Table 1). Sub-lineages 4.1.2.1 and 4.2.2.2 exhibited MIC values that matched the critical concentration for first-line anti-TB drugs, except for RIF, where the MIC was half of the critical concentration. In contrast, lineages 3 and 7 exhibited increased sensitivity to RIF and STM, needing only half of the standard critical concentration established for the microdilution broth method. Additionally, lineage 3 showed increased sensitivity to INH, requiring just half of the conventional critical concentration set for the same method.

## Discussion

We studied the growth kinetics of various *Mtb* strains in liquid 7H9 Middlebrook broth. Our findings reveal distinct growth patterns. All strains began growing on day 6, with sub-lineage 4.1.2.1 showing rapid exponential growth from day 9. This phase lasted about 6 days, after which all strains entered the stationary phase around day 15, likely due to a common growth-limiting factor like nutrient depletion. Sub-lineage 4.1.2.1 reached the highest bacterial concentration (*C*_max_), surpassing sub-lineage 4.2.2.2, lineage 7, lineage 3, sub-lineage 4.6.3, and the reference H37Rv strain. This suggests that sub-lineage 4.1.2.1 may have unique genetic or physiological traits for more efficient growth, and these differences in *C*_max_ could indicate varying abilities to adapt to environmental stress, such as nutrient availability or immune response factors in a host setting (Mashabela et al., 2019; Kanabalan et al., 2021; Saelens et al., 2019).

After entering the host’s airway, *Mtb* is taken up by alveolar macrophages, triggering an inflammatory response that promotes granuloma formation. This structure forms as immune cells are recruited to the infection sites, confining bacterial growth within macrophage phagosomes. *Mtb*’s capacity to survive and multiply within macrophages influences the quantity of bacteria released in respiratory droplets, enhancing its transmission potential and contributing to bacterial hypervirulence (Aiewsakun et al., 2021). The observed growth patterns in sub-lineages 4.2.2.2 and 4.1.2.1 particularly their high *C*_max_, rapid progression into exponential growth, or elevated bacillary concentrations may impact their higher virulence and wider transmission when compared to other *Mtb* sub-lineages. Sub-lineage 4.1.2.1 (Haarlem), is known for its significant global distribution (Negrete-Paz et al., 2021) and representing about 10% of *Mtb* cases in Ethiopia (Mekonnen et al., 2019), suggesting a competitive advantage in various host environments, potentially enhancing this sub-lineage’s ability to spread across populations. Conversely, sub-lineage 4.2.2.2 shows a more restricted geographical prevalence, notably in Ethiopia, where it accounts for approximately 15% of cases. This sub-lineage demonstrates heightened virulence and a propensity for drug resistance, which may be associated with genetic variations that might impact macrophage infection pathways and immune evasion mechanisms (Getahun et al., 2024).

Faster-growing strains could potentially lead to more aggressive infections, and understanding these differences at the growth kinetics level could inform tailored treatment approaches (Iacob and Iacob, 2013). Further research is needed to investigate the underlying genetic factors driving these variations in growth kinetics and their impact on disease progression and treatment outcomes in different *Mtb* lineages.

The multiple comparison study reveals significant growth differences among various *Mtb* sub-lineages, with sub-lineage 4.2.2.2 showing a notable growth advantage compared to all other strains from day 9 or day 12 until day 21. Similarly, sub-lineage 4.1.2.1 exhibited significantly higher growth rates or CFU counts when compared to H37Rv, sub-lineage 4.2.2.2, and sub-lineage 4.6.3 from day 9 onwards. These consistent growth advantages, and higher bacterial load suggest that sub-lineages 4.2.2.2 and 4.1.2.1 may possess adaptive traits that enhance their survival and replication, potentially increasing their pathogenicity, causing severe disease or resistance to host defenses and antibiotics (Thwaites et al., 2008; Tram et al., 2018).

In contrast, the other sub-lineages demonstrated inconsistent but significant differences in growth rates or CFU counts on various days, indicating that they may lack the same level of adaptability or resilience. No significant differences were found between H37Rv and sub-lineage 4.6.3, suggesting a closer relationship between these strains as observed in the phylogenetic tree (Figure 1). Overall, these findings emphasize the diversity in growth patterns among *Mtb* sub-lineages, which could have important implications for TB treatment and disease progression. Further research is needed to explore the genetic and phenotypic factors driving these differences.

The study emphasizes the differences in MIC values for first-line anti-TB drugs (RIF, INH, STM, and EMB) among various *Mtb* strains from lineages/sub-lineages commonly found in Ethiopia, even though all strains are susceptible to these drugs. Using the microdilution broth method, we observed distinct differences in drug susceptibility between various *Mtb* lineages. Sub-lineages 4.1.2.1 and 4.2.2.2 exhibited MIC values that were consistent with the critical concentrations set for first-line anti-TB drugs (except RIF), indicating a standard level of drug susceptibility in these sub-lineages. This suggests that these sub-lineages respond to treatment in line with the expected drug efficacy thresholds established by WHO guidelines.

In contrast, lineages 3 and 7 demonstrated significantly increased sensitivity to RIF and STM, requiring only half of the standard critical concentration for inhibition. This heightened sensitivity suggests that these lineages might respond more effectively to lower doses of these anti-TB drugs compared to other *Mtb* strains. Furthermore, lineage 3 also showed increased sensitivity to INH, with MIC values at half of the conventional critical concentration.

Lineage 3 has a prevalence of 21% in Ethiopia, with a 24.1% resistance rate to isoniazid. In contrast, lineage 4 has a prevalence of approximately 70% and a higher isoniazid resistance rate of 74.1% (Getahun et al., 2024). Among the sub-lineages, L4.2.2.2 is the most prevalent drug-resistant group, responsible for 50% of resistant cases and 34.5% of MDR^+^ cases (Mekonnen et al., 2023). Additionally, our findings indicate that lineage 3 has lower growth kinetics, bacillary load, and MIC values compared to sub-lineages 4.2.2.2, 4.1.2.1, and L7. The increased sensitivity of L3 to INH, STM and RIF may be influenced by either its lower bacillary load or the specific type of L3 strain circulating and examined in this study.

These findings underscore the importance of considering lineage-specific drug responses in TB treatment. Administering the same dose of antituberculosis drugs across different *Mtb* lineages may have significant implications for treatment effectiveness and resistance development (Chae and Shin, 2018). Different *Mtb* lineages exhibit distinct growth rates, virulence factors, and drug susceptibility profiles. For instance, faster-growing or more virulent lineages, such as those with high bacillary loads and rapid replication, may require higher or more frequent doses for effective clearance, as they might respond to drugs differently or evade host immune responses more efficiently. Standard dosing may be insufficient for such lineages, potentially leading to suboptimal drug exposure, which can promote resistance development and reduce treatment success.

It was reported that highly spreading and drug resistance, MDR, and XDR TB strains present in certain regions are linked to specific *Mtb* lineages, often confined to those areas (Chae and Shin, 2018; Malm et al., 2017). For example, lineage 4.2.2.2, which is geographically restricted, has demonstrated a heightened likelihood of drug resistance (Mekonnen et al., 2023; Getahun et al., 2024) and may not respond optimally to standard dosing due to its genetic adaptations. The one-size-fits-all dosing strategy might therefore undermine treatment efficacy in specific lineages, especially those more likely to develop resistance. Understanding host pathogen gene interactions and tailoring drug regimens to the characteristics of specific *Mtb* lineages could improve patient outcomes, slow resistance spread, and ensure the prolonged efficacy of current therapies.

While determining growth kinetics and drug sensitivity analyses of various *Mtb* lineages, the number of isolates considered for this analysis was limited. Thus, future research on larger sample set from each lineage would therefore be invaluable for confirming these findings on a broader scale. Another limitation of this study lies in the potential discrepancies between *in vitro* and *in vivo* conditions, which may affect the interpretation of growth kinetics and drug susceptibility data. *In vitro* models, while valuable for studying *Mtb* growth characteristics and drug responses, lack the complex interactions and immune pressures present in a host environment. For instance, *in vivo* conditions expose *Mtb* to immune responses, oxygen gradients, and nutrient limitations, which can alter bacterial metabolism and growth rates. These differences mean that findings from *in vitro* studies might not fully reflect the pathogen’s behavior within the human body, potentially impacting our understanding of how quickly certain lineages replicate or respond to drugs in real infections.

Furthermore, drug efficacy observed *in vitro* may not directly translate to *in vivo* outcomes, as host factors such as drug absorption, distribution, and immune-mediated effects play crucial roles in treatment effectiveness. These limitations highlight the need for caution when extrapolating *in vitro* findings to clinical scenarios and underscore the importance of complementing *in vitro* data with *in vivo* or clinical studies to better understand *Mtb* lineage-specific growth patterns and drug susceptibility. Moreover, while the study focused on first-line anti-TB drugs, it did not evaluate the effects of second-line or newer TB treatments. Understanding lineage-specific responses to a broader range of drugs could provide a more comprehensive view of treatment options. This study also did not explore the genetic mechanisms underlying the observed differences in growth kinetics and drug sensitivity between the various *Mtb* lineages. Future studies are warranted to further validate our observations, while addressing these limitations.

In conclusion, the findings from this study hold significant practical implications for improving TB treatment strategies in Ethiopia and comparable regions. By highlighting the growth characteristics, drug resistance tendencies, and geographic distribution of specific *Mtb* sub-lineages, our research suggests that tailored treatment approaches could improve therapeutic outcomes and mitigate the spread of drug-resistant strains. For example, considering lineage-specific drug dosing or developing diagnostic tools that quickly identify high-risk sub-lineages could allow for more targeted interventions.

To build on these findings, future research should include *in vivo* studies to validate the clinical relevance of these lineage-specific growth and drug resistance patterns. Additionally, large-scale epidemiological studies could further assess the distribution and transmission dynamics of these sub-lineages, while pharmacokinetic and pharmacodynamic studies might determine the most effective dosing strategies for each lineage. Ultimately, integrating these findings into public health policy and clinical practice could improve TB control efforts, particularly in areas where drug-resistant and virulent *Mtb* lineages are prevalent.

## Data Availability

The whole genome data from this study have been submitted to the NCBI repository under BioProject ID PRJNA1201357.
